# High rate of clinically unrecognized SARS-CoV-2 infections in pediatric palliative care patients

**DOI:** 10.1007/s00431-021-04242-5

**Published:** 2021-09-01

**Authors:** Benedikt Bötticher, Lars Dinkelbach, Martina Hillebrecht, Ortwin Adams, Oliver Dechert, Laura Trocan, Jennifer Neubert, Arndt Borkhardt, Gisela Janßen

**Affiliations:** 1 Department of Pediatric Oncology, Haematology and Clinical Immunology, Center for Child and Adolescent Health, Medical Faculty, Heinrich Heine University, Duesseldorf, Germany; 2 Institute of Virology, Medical Faculty, Heinrich Heine University, Duesseldorf, Germany

**Keywords:** Pediatrics, COVID-19, SARS-CoV-2, Comorbidities, Palliative care

## Abstract

Little is known about the frequency and clinical course of severe acute respiratory syndrome coronavirus 2 (SARS-CoV-2) infections in pediatric patients with severe comorbidities. In this prospective cross-sectional trial, the seroprevalence of SARS-CoV-2-IgG in patients with life-limiting conditions being treated by a large specialized pediatric palliative home-care team was determined. In order to gain insight into the infection chain, close contacts of seropositive patients were also included in the study. We analyzed the sera of 39 patients and found a 25.6% seroprevalence for SARS-CoV-2. No SARS-CoV-2 infections were known prior to the study. No significant difference was found in the symptom load between seropositive and seronegative patients during the risk period for SARS-CoV-2 infections. Of the 20 close contacts tested, only one was seropositive for SARS-CoV-2.

*Conclusions*: Our results indicate a substantially high prevalence of silent SARS-CoV-2 infections in pediatric palliative care patients. Surprisingly, no severe outcomes were seen in this fragile patient collective with severe comorbidities. The chain of infection and thus the reason for the high frequency of SARS-CoV-2 infections in pediatric palliative care patients remain unclear.

**Table:** 

**What is Known:** *•Even though severe disease courses of COVID-19 have been reported in children, there are yet no established risk factors for SARS-CoV-2 in pediatric patients.*	
**What is New:** *•In this cross-sectional seroprevalence study of palliative pediatric patients with severe life-limiting conditions, a high rate of seropositive patients (25.6%) was found.* *•Surprisingly, all seropositive patients were previously unrecognized, despite the severe comorbidities of our collective.*

**What is Known:**

*•Even though severe disease courses of COVID-19 have been reported in children, there are yet no established risk factors for SARS-CoV-2 in pediatric patients.*

**What is New:**

*•In this cross-sectional seroprevalence study of palliative pediatric patients with severe life-limiting conditions, a high rate of seropositive patients (25.6%) was found.*

*•Surprisingly, all seropositive patients were previously unrecognized, despite the severe comorbidities of our collective.*

## Introduction

There is still little knowledge about the course of coronavirus disease 2019 (COVID-19) and its prevalence in pediatric patients with severe or multiple comorbidities. Pediatric palliative care patients represent a heterogeneous group of patients with severe life-limiting conditions, of which neurological conditions, perinatal asphyxia, and malignancies are most frequent [1]. The global spread of COVID-19 and the resulting measures to curb this infectious disease have an impressive impact on the daily care of these patients [2]. With a large spectrum of severe underlying comorbidities, these patients form a potentially vulnerable cohort for severe courses of COVID-19. In order to gain further knowledge about the seroprevalence and course of COVID-19 in pediatric patients with severe comorbidities, we determined the serostatus and medical history of patients treated by a pediatric palliative home-care team. 

## Methods

This study was conducted on pediatric and young adult palliative patients aged 0.5 to 28 years in the period between 17 December 2020 and 19 February 2021. All patients were taken care of by the specialized pediatric palliative home-care team of the university hospital Duesseldorf, Germany. The team consisted of four nurses, four physicians, one psychologist, and one social worker. No specific allocations of healthcare workers to patients were made. Due to the rapidly progressive nature of end-stage malignancies, no patients with oncological diseases were included in the study.

Since the beginning of 2020, all healthcare workers of the pediatric palliative care team with contact to patients were routinely wearing FFP2 masks. In case of a supposed infection, healthcare workers were additionally wearing full protective equipment consisting of a face shield and work coats. The investigation took place before healthcare workers of the pediatric palliative care team received the full protective vaccination schedule. 

To determine the serostatus of our patients, SARS-CoV-2 IgG enzyme-linked immunosorbent assays were used (No. EI 2606–9601 G, EUROIMMUN, Lübeck, Germany). For sera tested before 15 February 2021, a signal/cutoff ratio of ≥ 1.1 was considered seropositive, a ratio ≤ 0.8 as seronegative. For patients included after 15 February 2021, a more recent test version using binding international units was used, and a value ≥ 35.2 was considered seropositive. The results of two samples were initially laid in the indeterminate region (signal/cutoff ratio 0.8–1.1); both sera were retested using binding international units and yielded seropositive results and thus were considered seropositive. In one of the patients in the indeterminate range, new material was collected 4 weeks after the initial testing and turned out negative.

Additionally, all participating families filled out a questionnaire assessing the patient’s medical history. The questionnaire covered (i) the frequency of COVID-19 symptoms and hospital admissions since February 2020, (ii) any exposure to known COVID-19 patients, and (iii) preexisting comorbidities and demographic variables (see Table 1). The questionnaire did not distinguish whether only one or multiple episodes of a symptom occurred within the given time frame. To identify differences between serologically SARS-CoV-2-positive and serologically SARS-CoV-2-negative pediatric palliative care patients in categorical variables, exact Fisher tests were calculated. For quantitative variables, Mann–Whitney *U* tests were used. *P*-Values below 0.05 (two-tailed) were considered statistically significant. Due to the explorative nature of this study, no adjustment for multiple comparisons was performed. The study was approved by the local ethical committee (Project-ID: 2020-1197) and performed in accordance with the Helsinki Declaration.

**Table 1:** Characteristics of SARS-CoV-2 seropositive and seronegative pediatric palliative care patients. Comparison of demographic variables, symptom load and known COVID-19 contacts in SARS-CoV-2 seropositive and seronegative pediatric palliative care patients. Symptoms were retrospectively assessed via interview. A direct contact was defined as a direct close-distance interaction for > 15 min between the patient and the person who tested positive for COVID-19. A low-risk contact was defined as direct interaction between the patient and the person who tested positive for COVID-19 for a shorter time period or at a longer distance. An indirect contact was defined as direct contact to a member of the same household, e.g. the patient’s parents

	Seropositive		Seronegative		*p*-value
	10	(25.6%)	29	(74.4%)	
**Demographics**					
Age	10.0	± 7.8	12.2	± 7.6	0.46
Gender (male)	7		20		1.00
**Diagnoses**					
Non-neurological conditions	1		1		0.14^*^
Progressive neurological conditions	7		12		
Severe neurological impairment with intermittent aggravations	2		16		
**Symptoms from February 2020 to inclusion into the study (time frame: 17.12.2020–19.02.2021)**					
Patients admitted to hospital	6	(60.0%)	14	(48.3%)	0.72
Patients with an episode of fever (> 38.5 °C)	9	(90.0%)	16	(55.2%)	0.06
Duration of fever episode (days)	5.6	± 6.2	4.1	± 3.4	0.63
Maximum temperature (°C)	39.6	± 0.6	39.4	± 0.63	0.69
Coughing	6	60.0%)	16	(55.2%)	1.00
Dyspnea^a,b^	4	(44.4%)	15	(53.6%)	0.71
Oxygen supplementation^c^	7	(70.0%)	13	(48.1%)	0.29
Non-invasive ventilation	3	(30.0%)	4	(13.8%)	0.34
Invasive ventilation	0	(0.0%)	5	(17.2%)	0.30
Rhinorrhoea^b^	3	(33.3%)	18	(62.1%)	0.25
Diarrhea	3	(30.0%)	15	(51.7%)	0.29
Emesis	7	(70.0%)	14	(48.3%)	0.29
**Known COVID-19 contacts**					
Direct high-risk contact	1	(10.0%)	2	(6.9%)	1.00
Direct low-risk contact	1	(10.0%)	1	(3.4%)	0.45
Indirect contact	0	(0%)	5	(17.2%)	0.30

Due to missing values

^*^The *p*-value refers to a 2 × 3 exact Fisher test including all three diagnosis groups

^a^only 28 seronegative and/or

^b^only 9 seropositive patients or

^c^only 27 seronegative were included in the analyses of this variable

## Results

Of 61 patients and their families being cared for during the study period, *n* = 39 agreed to participate in this study (mean age 11.6 ± 7.6 years, 27 male). Table 1 depicts the demographic and clinical characteristics of all included patients. Of the included patients, ten (25.6%) were seropositive for IgG antibodies specific to SARS-CoV-2. None of those seropositive patients had a known SARS-CoV-2 infection prior to our study. In seven of those ten patients, at least one nasopharyngeal swab for SARS-CoV-2 had been obtained since February 2020, prior to hospital or hospice admission or after the presentation of respiratory symptoms. None of those swabs turned out positive.

No significant differences in demographic variables or underlying diseases were found between seropositive and seronegative patients (see Table 1). In the retrospectively collected medical histories, no significant differences were detected in the SARS-CoV-2-specific symptom load or regarding the frequency of contact with known COVID-19 patients since February 2020.

Seven out of ten seropositive patients suffered from severe encephalopathies (Mitochondriopathy, Zellweger syndrome, Ohtahara syndrome, Lennox–Gastaut syndrome, Aicardi–Goutières syndrome, Morbus Canavan, and severe cerebral atrophy of unclear genesis); one patient suffered from spinal muscular atrophy type 1; one patient from cystic fibrosis; and one patient from nonketotic hyperglycinemia.

Since February 2020, six seropositive patients had been admitted to the hospital, and five of those patients had had multiple hospital admissions. One patient with cystic fibrosis who had been dependent on non-invasive ventilation since 2017 had had two pulmonary exacerbations requiring hospital admissions, both times testing negative for SARS-CoV-2 using PCR. One patient with Ohtahara syndrome had been admitted to the hospital during a rhinovirus-confirmed respiratory infection and became temporarily dependent on non-invasive ventilation. A patient with Morbus Canavan had been admitted to the hospital due to a respiratory infection of unknown origin (nasopharyngeal SARS-CoV-2 swab was negative). The patient with spinal muscular atrophy had had two hospital admissions for intrathecal administration of nusinersen. He had also been dependent on non-invasive ventilation since 2017. Two seropositive patients had had hospital stays for minor surgical procedures, and two had been admitted to the hospital due to increases in epileptic seizures.

In seropositive patients, close contacts (relatives, nurses, or similar) were asked to participate in this study. Of the 20 close contacts tested (16 parents, two adult siblings, one grandmother, one nurse), only one mother was seropositive for SARS-CoV-2.

## Discussion

In this sample of pediatric palliative care patients, we found a high seroprevalence of SARS-CoV-2-specific IgG of 25.6%. Surprisingly, no critical or fatal disease courses were seen, despite the severe multiple comorbidities of those patients. Compared to available data for the seroprevalence of SARS-CoV-2-specific antibodies in the German population, this is nearly nine times higher [3]. However, these datasets mostly include data about adult subjects collected in the first half of 2020 and are therefore only comparable to a limited degree. Nailescu et al. found a low seroprevalence in pediatric kidney transplant recipients at an American kidney transplant center [4]. The authors attributed the low seroprevalence to the social distancing practiced by the patients and their families [4]. However, comparing the timeframes of the studies (May–June of 2020 vs. December 2020–February 2021), the ongoing spreading of SARS-CoV-2 may have resulted in a higher seroprevalence in our sample, despite social distancing measures.

Still, considering the low prevalence (5%) of SARS-CoV-2-IgG in tested relatives, potential sources of SARS-CoV-2 infection in our pediatric palliative collective have to be discussed. There is reasonable evidence of high seroprevalence and asymptomatic viral carriage in healthcare workers [5], making them a potential source of infection. However, frequent contact to nursing services, special therapists, and regular visits from our palliative home-care team is essential for providing our patients with the best possible care.

Counterintuitively, there were no known COVID-19 cases in this study collective prior to this study, despite the high seroprevalence of SARS-CoV-2 in our potentially vulnerable patients with severe comorbidities. Since February 2020 to inclusion into the study, six of the seropositive patients had to be admitted to the hospital, and three were at least temporarily dependent on non-invasive ventilation. However, none of these events was likely related to a SARS-CoV-2 infection, and it is more probable that they reflect the generally fragile state of our patients. Nevertheless, even though negative nasopharyngeal swabs were collected on multiple occasions, false-negative results cannot be ruled out. Statistically, no difference was detected in the symptom load between seropositive and seronegative patients. However, due to the low sample size of our study, actual differences in the symptom load between seropositive and seronegative patients might have been missed. Additionally, a potential recall bias might have confounded our results given the long retrospective period of our study.

In adults, several comorbidities, such as cardiovascular disease, diabetes, obesity, and chronic pulmonary diseases, have been identified as risk factors for a critical course of COVID-19 [6]. Even though severe disease courses of COVID-19 have been reported in children [7, 8], there are yet no established risk factors for hospitalization or mortality related to SARS-CoV-2 in pediatric patients. In a study of PCR-positive pediatric patients, preexisting neurologic, cardiac, and hemato-oncologic conditions were associated with a higher likelihood of hospitalization due to COVID-19 [9]. However, these comorbidities did not differentiate between a critical and non-critical course of COVID-19 in hospitalized patients, so the reason for hospitalization may have been the underlying condition and the resulting fear of disease progression, and not the severity of COVID-19 per se. Despite severe comorbidities in pediatric palliative patients and their low resilience to respiratory infections, all cases of COVID-19 in our study remained clinically unrecognized. Besides comorbidities, genetic alterations in the ABO blood group system and variants of the 3p21.31 gene cluster have been linked to susceptibility to COVID-19 disease progression in adults [10]. In a case series of four young male patients, X-chromosomal loss-of-function variants of the gene encoding for Toll-like receptor 7 were associated with severe COVID-19 [11]. All patients became dependent on ICU care, and one patient died, despite good preexisting general health conditions [11]. Genetic alterations as monogenetic errors of the immune system may be the main driver for severe disease progression in COVID-19, especially in young patients [12]. Ongoing monitoring and genome-wide association studies are needed to gain further insight into the disease course and risk factors for severe SARS-CoV-2 infections in pediatric patients.

## Conclusions

Pediatric palliative care patients have a high risk for SARS-CoV-2 infections. Surprisingly, all cases of COVID-19 in our sample remained clinically unrecognized, despite severe multiple comorbidities. Genetic predispositions may be of greater relevance to the clinical course of COVID-19 in young patients than general health conditions [11].

## Data Availability

Data may be available upon request by contacting the corresponding author.

## Abbreviations

COVID-19: Coronavirus disease 2019
SARS-CoV-2: Severe acute respiratory syndrome coronavirus 2
